# Genetic Determinants of Human Health Span and Life Span: Progress and New Opportunities

**DOI:** 10.1371/journal.pgen.0030125

**Published:** 2007-07-27

**Authors:** George M Martin, Aviv Bergman, Nir Barzilai

**Affiliations:** Johns Hopkins University School of Medicine, United States of America

## Abstract

We review three approaches to the genetic analysis of the biology and pathobiology of human aging. The first and so far the best-developed is the search for the biochemical genetic basis of varying susceptibilities to major geriatric disorders. These include a range of progeroid syndromes. Collectively, they tell us much about the genetics of health span. Given that the major risk factor for virtually all geriatric disorders is biological aging, they may also serve as markers for the study of intrinsic biological aging. The second approach seeks to identify allelic contributions to exceptionally long life spans. While linkage to a locus on Chromosome 4 has not been confirmed, association studies have revealed a number of significant polymorphisms that impact upon late-life diseases and life span. The third approach remains theoretical. It would require longitudinal studies of large numbers of middle-aged sib-pairs who are extremely discordant or concordant for their rates of decline in various physiological functions. We can conclude that there are great opportunities for research on the genetics of human aging, particularly given the huge fund of information on human biology and pathobiology, and the rapidly developing knowledge of the human genome.

## Introduction

Other articles in *PLoS Genetics* and elsewhere have documented remarkable progress in genetic aspects of aging in model organisms. These studies have revealed what can be regarded as the first “public” mechanism of aging, that is to say, a biochemical genetic pathway, modulation of which can alter the life spans of diverse species [1–3]. We applaud the remarkable achievements of our colleagues working with these models. One of us was in fact an early champion of this approach [4]. These model systems have several limitations, however. First, the life spans of these models are dramatically shorter than those of humans. The mutations that extend life span result in increases of a few weeks or months in invertebrates and a year or so in rodents. These species are “r” selected species, characterized, in part, by rapid rates of development, high degrees of fecundity, and short life spans [5]. Human beings are “K” selected organisms, characterized, in part, by long periods of development, comparatively few progeny and long life spans [5]. It is therefore possible that the biochemical genetic results from model organisms may not be relevant for humans, whose life history strategies are quite different. It should be noted, for example, that a polymorphic locus *(CETP)* that modulates susceptibility to several major diseases of aging does not exist in invertebrates or rodents. Second, while we have learned a great deal about developmental biology from worms, flies, and mice, there is a paucity of detailed information on the pathophysiology of aging and of their variations among genetically heterogeneous wild-type populations, particularly in worms and flies. In contrast, there is a vast literature on these and all other aspects of human biology, including remarkable progress in human genetics (Table 1). Moreover, physicians have provided detailed characterizations of late-life disabilities and diseases in human populations (Table 2). Third, additional and unique DNA sequences have evolved in *Homo sapiens,* including rapidly evolving functionally significant intronic sequences that distinguish us from our nearest relative, the common chimpanzee *(Pan troglodytes),* whose life span is approximately half that of humans) [6,7] (see, however, evidence of a single outlier who lived to age 74, http://genomics.senescence.info/species/biblio.php?id=0505). Fourth, most gerontological investigations of model organisms have utilized highly inbred organisms typically examined in a single environment. By contrast, human geneticists, particularly medical geneticists, see the results of a huge range of gene–gene and gene–environmental interactions. They therefore have considerable opportunities to contribute to our understanding of why individual patterns of aging exhibit such substantial variations. Some of this understanding should prove to be unique to our species, as they will include “private” biochemical genetic mechanisms—that is to say, mechanisms that are characteristic of only particular subsets of individuals [1–3]. These different patterns of aging certainly include various degrees of susceptibility to both common and rare late-life diseases and disabilities. As we shall see below, these are all part of a spectrum of phenotypes that escape the force of natural selection and are thus, in our view, part and parcel of complex aging processes. We can refer to them generically as “senescent phenotypes” [8]. Moreover, given the likelihood that intrinsic physiological declines in structure and function are early precursors of such geriatric disorders, investigators have the opportunity to work backwards from the disease to elucidate underlying mechanisms of aging, mechanisms that set the stage for the emergence of these clinically important disorders. Finally, studies of genetic contributions to late-life disorders can elucidate variations in health span, which can be defined as the period of life during which an individual is free of chronic illness and substantial functional decrements. Genetic and epigenetic factors that limit health span are certainly legitimate aspects of biogerontological research, particularly from the point of view of medical economics. A cogent example of why long life span cannot be equated with long health span comes from studies of pedigrees with certain forms of autosomal dominantly inherited frontotemporal dementia. Affected subjects may exhibit up to a 26-year history of personality disorders, cognitive decline and, eventually, overt dementia [9]. The underlying mutation in these particular pedigrees involves mutations at the tau locus, leading to a greatly accelerated rate of accumulation of neurofibrillary tangles, lesions that are commonly observed, in much smaller numbers, in the brains of much older individuals with wild-type versions of that gene. (Studies of different pedigrees with frontotemporal dementia have recently documented causal mutations at a second locus, progranulin *[PRGN],* the product of which is a glycoprotein that probably functions as a secreted growth factor [10]). We shall give below only a few other examples of how disorders listed in Table 2 have led to molecular understanding of certain common forms of pathophysiology in aging human subjects, thus explaining some of the variations in senescent phenotypes.

**Table 1:**
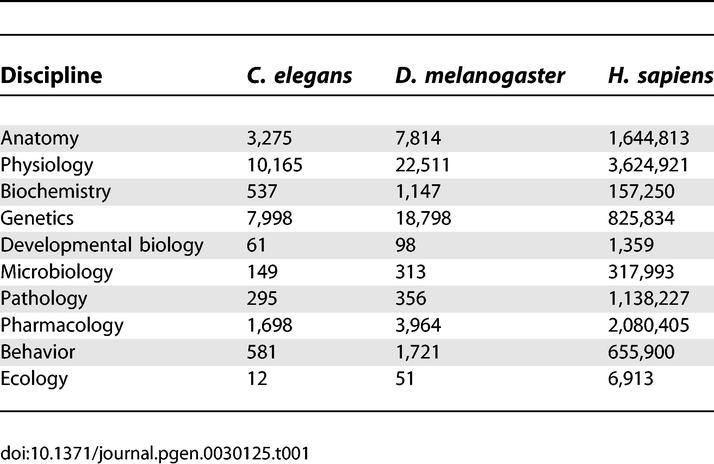
Some Reasons Why Homo sapiens Will Become THE model Organism of the 21st Century: PubMed Citations from 1950 to May 2006

**Table 2:**
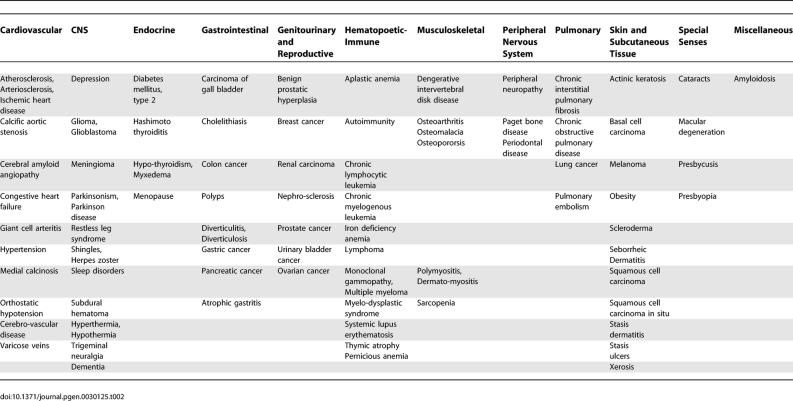
A Tabulation of Major Geriatric Disorders According to the Body Systems That Are Primarily Affected

Human geneticists have only recently begun to use the tools of linkage analysis and association studies to identify alleles contributing to exceptionally long life spans. Interesting candidates have emerged from these studies. There is virtually no information, however, on the genetic basis of differential rates of decline in well-defined physiological functions among human populations. We shall argue that such studies, particularly those that are initiated in middle age, before the onset of complicating comorbidities, should have a high priority for research, as they would have the potential to discover genes that impact on rates of aging within various organ systems. Such investigations, moreover, would serve as tests of the null hypothesis that there is significant asynchrony in rates of physiological aging among major body systems in human subjects. Selective pressures can be expected to maintain a degree of synchrony in the rates at which various organ functions decline [11,12], but that synchrony is not exceptionally tight, as is evident from clinical observations of marked variations in patterns of senescence. The pursuit of such investigations in human populations has numerous advantages, not the least of which is that they are so relevant to the human condition, particularly with regard to higher cognitive functions.

## An Evolutionary Biological Approach to Understanding Gene Action in Human Aging

The classical evolutionary biological theory of aging tells us that senescence occurs in age-structured populations because of the decline in the force of natural selection with age [11–13]. That generalization has been challenged recently [14]. Certain species of fish, for example, continue to grow throughout their life spans; for such species, rates of aging may be “negligible” [15,16] and the force of natural selection could conceivably increase with age [17]. No human being has ever exhibited such a life history, however. All physiological studies have confirmed gradual functional declines in multiple body systems beginning at middle age, even for cohorts of exceptional athletes [18]. A wide variety of diseases and disabilities accompany these physiological declines.

It is not generally appreciated that the evolutionary theory of why we age provides clues to how we age [19–21]. Classes of such gene actions include: (1) “longevity assurance,” genes that enhance structure and function of the organism throughout the life span; (2) “antagonistic pleiotropy,” alleles selected because of enhanced reproductive fitness early in the life span, but with negative effects late in the life span, when those effects will have escaped the force of natural selection; and (3) “mutation accumulation,” constitutional mutations that do not reach a level of phenotypic expression until late in the life span—once again, when they will have escaped the force of natural selection, and thus could not be purged from the population. The evolutionary biological theory of aging predicts a polygenic basis for the control of rates of aging. While single gene variations can indeed enhance the life spans of model organisms, these involve the tweaking of diapauses [22,23], biochemical genetic pathways that most certainly did not escape the force of natural selection, as they were designed to enhance reproductive fitness by down-regulating reproductive activity and protecting the soma during transient environmental challenges. As such, they will eventually be trumped by a variety of gene actions that are predicted by the evolutionary biological theory of aging [21]. As discussed below, evolutionary ideas can also be borrowed and applied to one or two overlapping generations as an aid to the discovery of genotypes modulating longevity and age-related diseases.

## The Search for Polymorphisms and Mutations Modulating Susceptibility to Late-Life Disorders

Table 2 lists 87 disease and disability phenotypes that commonly emerge in geriatric subjects. These result in a substantial proportion of the overall morbidity and mortality in the developed societies and are responsible for a major proportion of the huge costs of Medicare in the United States. These costs are likely to increase substantially, given demographic trends [24]. A very large number of genetic variants that advance the ages of onset and/or the rates of progression of these phenotypes can be found by online searches of Online Mendelian Inheritance in Man (http://www.ncbi.nlm.nih.gov/sites/entrez?db=OMIM). These have the potential to reveal pathogenetic mechanisms. They can be divided into two major categories. One subset consists of mutations that impact upon a number of such phenotypes; they have been referred to as “segmental progeroid syndromes” [25]. A second group consists of allelic variants that impact predominantly on only a single tissue or organ system; these have been referred to as “unimodal progeroid syndromes” [26]. We should be alert, however, to the possibility that putative unimodal syndromes may in fact have more widespread, systemic effects; a striking example has been uncovered for the case of a mouse model of Huntington disease, in which a metabolic abnormality of brown fat and a defective regulation of body temperature were found to be associated with the triplet repeat sequences previously though to have an exclusive impact upon the central nervous system [27]. Striking examples of segmental and unimodal progeroid syndromes have been recently reviewed [8,28]; these have indeed provided important insights into mechanisms of aging—for example, the role of genomic instability for the case of many segmental disorders and the role of abnormal protein aggregates for the case of various unimodal dementing disorders. No single locus has ever been discovered, however, that appears to accelerate the rates of onset and/or the rates of progression of all senescent phenotypes [25]. Patients with Werner syndrome (WS), caused by mutation at a member *(WRN)* of the RecQ family of helicases [29], exhibit accelerating rates of development of all forms of arteriosclerosis, type 2 diabetes mellitus, gonadal atrophy, skin atrophy, hair loss and hair greying, regional loss of subcutaneous tissue, osteoporosis, ocular cataracts, and neoplasia [25]. While deserving of greater study, there is so far no convincing evidence of an acceleration in the rates of development of synaptic loss, beta amyloidosis of blood vessels or parenchyma, granulovacuolar degenerations, or neurofibrillary lesions [30], markers that accumulate, to varying degrees, in the aging brains of many aging human subjects, with or without a clinical or neuropathological diagnosis of Alzheimer disease. Those lesions are very abundant in that disorder, which exhibits exponential increases after the age of 65, with prevalence rates 25%–48% for persons over age 85 [31]. There are additional interesting discordances between the phenotype of WS subjects and what is commonly found in “usual” or “normative” aging. For example, the osteoporosis of WS is disproportionately severe in the bones of the lower limbs rather than in the vertebral bodies [25] and the patterns of neoplasia are quite unusual, as WS patients exhibit a high prevalence of sarcomas and rare neoplasms [32]. These are among the reasons for referring to such syndromes as “progeroid” (“like” premature aging). On the other hand, as noted above, the biochemical genetic findings are consistent with a growing body of evidence implicating genomic instability as a common basic mechanism of aging [33,34]. Somatic cells from subjects with WS exhibit marked accelerations in the rates of replicative senescence of several somatic cell types [35]. The discovery that G quartet motifs at telomeres are among the favored substrates for the *WRN* locus [36,37] is consistent with the important role of telomere loss as a mechanism leading to the replicative senescence of somatic cells [38]. WS has also pointed to the importance of aberrations in DNA transactions within the lens epithelial cells as an important mechanism of cataractogenesis, as opposed to post-translational alterations of lens crystallins, an alternative pathogenetic mechanism [39]. Patients with WS and the Hutchinson-Gilford Progeria Syndrome (HGPS), as well as a number of other progeroid syndromes, exhibit an accelerated loss of somatic cells, consistent with the widespread atrophy one observes in senescent human subjects. Finally, an important argument for a commonality of mechanisms of aging in HGPS and usual aging is that the splice variant caused by the common HGPS mutation, which functions as a dominant negative, thus impairing lamin A structure and function [40], also appears in the aging tissues of normal humans [41]. This may explain the appearance of comparable nuclear morphological abnormalities in the aging somatic cells of normal individuals, albeit at lower frequencies than that observed in the cells of HGPS patients [41].

A recent example of encouraging progress in the discovery of unimodal progeroid syndromes was the discovery of two polymorphic loci, complement factor H and a predicted gene on Chromosome 10q, LOC387715, that modulate susceptibility to age-related macular degeneration [42–44]. A prospective study of US nurses and health professionals has revealed a ∼50-fold increase (95% CI: 10.8–237) in the risk of age-related macular degeneration for subjects who are homozogous for both risk alleles [45]. Smoking and obesity increased the risks associated with these variants [45]. Of interest is the lack of evidence for an association with complement factor H in a Japanese population [46], but the presence of an association in this population with the polymorphism at the 10q locus [47]. Enormous efforts have also been directed to families with both early- and late-onset dementias of the Alzheimer type. All three autosomal dominant genes responsible for the comparatively early-onset variety impact upon the processing of the beta amyloid precursor protein, the result being increased proportions of the highly amyloidogenic amyloid beta 1–42 peptide (for a brief and selective review of this huge literature, see [48]. These findings are consistent with a major etiological role of amyloid beta peptides in the vastly more common late onset cases, where the neuropathological diagnosis is made on the basis of amyloid plaques and neurofibrillary tangles. With the exception of the important risk factor of the epsilon 4 allele at the *APOE* locus (reviewed by [49]), numerous candidate loci remain to be fully validated as contributors to the much more common late onset forms of late-life dementia. For a comprehensive list of these candidate loci and their polymorphic alleles, see http://www.alzforum.org/res/com/gen/alzgene/default.asp.

## Linkage and Association Studies of Exceptional Human Longevities

Centenarians have outlived any exceptionally long-lived invertebrate model by ∼100 years and any comparable rodent model by ∼30-fold. Human subjects in general and centenarians in particular outlive their nearest relatives, the common chimpanzee, by many decades [50]. This happy state of affairs is clearly the result of evolutionary changes in our constitutional genomes [51]. Since the structure of our proteins and those of chimpanzees is very nearly identical [52], our enhanced life spans are probably related primarily to regulatory RNA species, an area of scholarship that has just begun [6,7] and has not yet been applied to the study of the evolution of varying longevities.

Our task here, however, is to review progress towards the elucidation of genetic factors that contribute to exceptional longevities of individual members of *Homo sapiens.* The life expectancies of centenarians at birth are nearly double that of most members of their birth cohort and, on average, have surpassed current life expectancy by 22 years. Environmental and stochastic contributions to human life span likely play important roles in the determination of such exceptionally long survivals, as inferred from the twin studies discussed below. Familial aggregates of exceptional longevity do not rule out major environmental factors that are the result of cultural inheritance (e.g., lifestyles, nutrition); such factors could explain, in part, why the progeny of long-lived members of the Framingham study exhibit advantageous cardiovascular risk profiles in middle age [53]. Nevertheless, evidence consistent with a significant heritable component of exceptional longevity is impressive. Parents of centenarians (born in ∼1870) were shown to have approximately nine times the odds of living to the tenth decade as compared to controls [54]. Siblings of centenarians were shown to have up to an ∼18-fold increase in the chance of achieving a similar age [55]. Such data have raised the possibility that some specific genetic modulators of aging in humans can be identified using such populations, and that conserved pathways for exceptional longevity might thus be validated. Exceptional longevity is obviously coupled with exceptional resistance to diseases that lead to earlier mortalities. We do not have the required biomarkers, however, to clearly disentangle the two phenomena. The research suggested in the last section of this essay may eventually lead to such markers, however.

During the last decade, centenarian populations (New England American, Mormon, Ashkenazi Jewish, Islandic, Okinawan, Japanese, Italian, Irish, and Dutch, among others [54,56–60] have been used for association studies to search for candidate longevity genes or pathways. Particularly striking examples have included *PON1* [61–67] *IGF-1* [68–71], *PAPR-1* [72,73], cytokines, enzymatic antioxidants such as superoxide dismutases [74,75], and elements of lipid metabolism [76,77]. Some significant differences have been noted between younger cohorts and centenarians in the prevalence of specific genotypes and sometimes in their associated protein activities. These interesting observations, however, have suffered and will continue to suffer from several limitations. In addition to the usual problems and pitfalls of association studies, particularly as we enter the new age of whole genome scans [78], there is the special problem of the identification of appropriate controls for a cohort of exceptionally long-lived individuals. One innovative approach has been an experimental design based upon a genetic analysis of the progeny of centenarians, giving the opportunity for matched spousal controls [79].

The New England Centenarian Study recruited long-lived sib-ships for a genome-wide scan of linkage to exceptional longevity. A region on Chromosome 4 was implicated [80]. By high density SNP analysis an exonic genotype in microsomal transfer protein was thought to be the locus associated with the exceptional longevity [81]. The original finding could not be replicated in independent populations [82]. Such validation is crucial because of the considerable rates of false positives. While it is possible that the role for this gene in longevity may only be significant in certain populations, the most likely explanation for the original linkage was population stratification. The ethnic mix within the long-lived and younger control populations was likely to have differed [83]. Nevertheless, it would be helpful to evaluate other allelic variants in the same gene or in other related genes. In any case, this early study emphasizes the need to establish additional phenotypes associated with the polymorphism. Although microsomal transfer protein cannot be directly measured, evidence for a role in lipoprotein characteristics or a relationship to age-related diseases would have been helpful in support of a protective role. The population stratification problem can be ameliorated by the selection of better-defined populations, as was done for the case of the Dutch study cited above [83].

**Figure 1:**
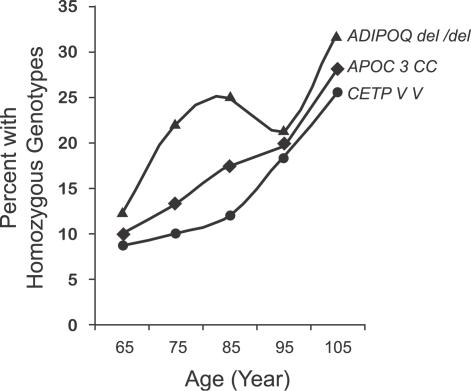
Visual Presentation of the Frequency Trends of Favorable Genotypes with Exceptional Longevity This trend was obtained in ∼400 Ashkenazi Jewish subjects over age 95 and ∼600 subjects between ages 60–95 [76,87]. While these genotypes were assessed cross-sectionally in groups between ages ∼60–110, it is important to realize that marked selection occurs during the life course. One also should be aware of the fact that very few subjects achieve centenarian status. Of many polymorphic candidate loci, only subjects homozygous for CETP VV, APOC-3 CC, and ADIPOQ del/del genotypes are markedly and significantly enriched among centenarians (see details in text). To be considered a favorable longevity genotype, a monotonic increase should be observed among age groups. This criterion helps to exclude false-positive associations that occur only in one age group but that do not exhibit trends among sequential age groups. Genotypes with unchanged frequencies among age groups serve as partial controls for genotypic distribution and stratification tests. The analysis of such patterns is useful for the identification of candidate “longevity genes.”

Studies performed at the Albert Einstein College of Medicine were based upon populations of Ashkenazi Jews and the following considerations [84]. First, exceptional longevity is obviously a rare phenotype (∼1/10,000 individuals live to the age of 94–110). Second, it is also apparent that, for any given cohort, genotypes associated with comparatively early mortality are “weeded out,” while a subset of genotypes are associated with survival. Given large cohorts representing each decade of the life span, one can examine whether those who continue to survive exhibit biologically distinctive phenotypes and genotypes as compared to those of younger cohorts. Thus, the relative prevalence of favorable “longevity” genotypes within the population can be expected to rise monotonically rather than abruptly or intermittently over the life course. Because the genotypes of survivors are “selected,” the greater the attribution of a genotype to longevity, the greater is the divergence from Hardy–Weinberg equilibrium among the elderly. Using this strategy, the Einstein group recruited significant numbers of Ashkenazi Jews of all ages, including ∼400 individuals between ages 95–110. Significant increases within aging cohorts were observed for three genotypes from among hundreds of candidate genotypes (selected because of their relevance for lipoprotein phenotypes) that were tested in unrelated populations consisting of individuals between ages 50–110 years (Figure 1) [79,85]. These genotypes were: (1) the *CETP* gene codon 405 isoleucine to valine variant *(CETP VV);* (2) the apolipoprotein C-3 (*APOC-3*) gene codon A (−641) C variant *(APOC-3 CC)*; and (3) a deletion at +2019 in the adiponectin *(ADIPOQ)* gene. The enrichment of the *CETP* genotype is supported by evidence from two independent populations [77,86].

**Figure 2:**
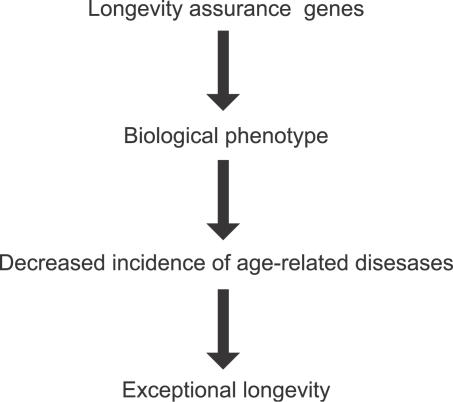
The Stages Needed in Order to Support the Association of a Genotype with Longevity While Figure 1 demonstrates how to obtain genotypes in genes that are important for the assurance of longevity, the verification of such genotypes requires additional analyses. First, one should seek evidence of a relevant biological phenotype. These may include in vitro and in vivo functional assays that demonstrate appropriate alterations in genes and the determination of plasma or tissue levels of substances that reflect an intermediate phenotype. Second, one should ideally develop lines of evidence demonstrating that a given genotype is protective against common diseases of aging, i.e., that the genotypes also modulate health span. These various steps should help to establish genetic factors contributing to exceptional longevity. As such, they should serve as major clues to the pathogenesis of common diseases of aging, thus providing rational strategies of prevention (see text).

While a significant overrepresentation of a single genotype among nonagenarians and centenarians operationally defines a candidate gene, several other criteria should be fulfilled before considering it to be an important longevity assurance gene (Figure 2).

The first step by which the functionality of the genotype can be studied is by determining the serum and plasma levels of the coded protein, if it is secreted and circulating. For example, for each of the genotypes in Figure 1 *(CETP*, *APOC3*, and *ADIPOQ),* appropriate alterations in plasma levels have been demonstrated [76,85–89]. Detailed information on the approach to the choice of controls typically used for these studies is given in [87]. They included spouses or other nonrelated age-matched pairs for the progeny of centenarians. The genomes of the latter can be expected to be enriched with alleles for unusual longevity. Indeed, these offspring were shown to be healthier than age-matched controls and had more favorable lipid profiles.

A second step in establishing functionality is the identification of an intermediate phenotype. For example, alleles at *CETP* and *APOC3* differentially modulate lipoprotein characteristics. These effects may vary with age and should therefore be examined in cohorts of varying ages. The functional value of a genotype can also be assessed directly by functional studies of the mutant in a cellular system in vitro*.* Such studies may underestimate or overestimate the real physiological importance of the relevant gene action in vivo, however. If the gene encodes a protease, then studies of its specificity, tissue distribution, and regulation are called for. If the gene encodes a cell-surface receptor, then studies of the biochemistry of the receptor should be done. For example, the Einstein group recently identified novel mutations in the IGF-1 receptor of three centenarians. AKT phosphorylation was assessed in lymphoblastoid cell lines from these subjects and controls, both basal and induced levels (stimulating with IGF-1). A marked decrease in phosphorylation was observed in cells from the centenarians with the mutant IGF-1 receptors [89,90].

As noted in the Introduction, the enhancement of life spans in model organisms via single gene mutations raises the question of whether allelic variations at this pathway in human subjects might impact intrinsic biological aging within all tissues and thus lead to substantial increases in life span. Among the many billions of human beings who have lived since the time of recorded history, it is unlikely that a spontaneous mutant of this type, say leading to a doubling of the usual human life span, would have been missed. One could not rule out gene actions of this type, however, that contribute to the generation of rare, relatively healthy centenarians. This possibility is supported by at least three lines of evidence. First, as in the case of *CETP VV,* the protection provided by certain genotypes can extend beyond a known role in a disease entity associated with its ascertainment (in this example, cardiovascular disease). The *CETP VV* genotype is also associated with enhanced insulin sensitivity and lower risk for hypertension, the metabolic syndrome, and diabetes [87]. Moreover, the *CETP VV* genotype protects against age-related cognitive decline and Alzheimer's disease [91], although the role of particular haplotypes at that region may interact with polymorphic alleles at the *APOE* locus [92].

A second example is a 2.5-fold increase, among centenarians, in the prevalence of an apoC III promoter variant. This variant is associated with significant declines in plasma levels of apoC III and a phenotype of large lipoprotein particles. There is also significantly less hypertension among subjects homozygous for this variant. The most striking data, however, were obtained from a retrospective study of a cohort of subjects bearing this variant. They live significantly longer; in fact, subjects <95 years old with this genotype lived on average over four years longer than those who were not homozygous for the variant. This is indeed a very large impact upon life span when one considers the conclusions of demographers, who have noted that the elimination of ischemic heart disease, a disorder that was responsible for 25.73% of all deaths in 1985, would increase life expectancy at birth by only 3.0 years for females and 3.55 years for males [93].

A third argument suggesting that some longevity genes are not merely disease specific is the marked conservation of some of these loci. For example, apoC III is under the control of *FOXO-1,* a transcription factor homologous to the *DAF16* gene of *Caenorhabditis elegans. DAF16* is a key regulator of a downstream suite of genes that are thought to protect the organism from macromolecular damage and thus enhance life span [94]. Some centenarians have novel functional mutations in the IGF-1 receptor, as noted above. There is evidence that the homologue in mice regulates life span and resistance to oxidative stress [95]. Thus, while the impact of variants at the *CETP* locus upon age-related diseases and longevity may be a special feature of the biology of humans, there is also evidence that the fruits of research on the genetic modulation of the life spans of worms, flies, and mice may in fact be applicable to our species.

Some favorable “longevity genotypes” may act to buffer the deleterious effects of genes that lead to age-related diseases. As a result, the frequencies of deleterious genotypes may, paradoxically, be increased among individuals with extreme life spans. This may explain why the cholesteryl ester transfer protein (CETP-VV) genotype appears to exhibit an additional advantageous effect—the neutralization of the deleterious effects of the lipoprotein(a) (Lp(a)) gene [84]. Such buffering effects cannot be ascribed to genetic linkage. For the example just cited, those loci are in fact on separate chromosomes. More generally, however, it is clear that one can define two distinct populations, each bearing the disease susceptibility allele in question, but only one of which exhibits the putative buffering effect.

## The Role of Stochastic Events in the Modulations of Health Span and Life Span

A study of uncensored pairs of Danish human twins has indicated that only about one quarter of the heritability of life span can be attributed to the constitutional genotype [96]. There are indications from twin studies of very old individuals, however, that more robust genetic contributions to superior health and superior cognitive functioning can be identified [97,98]. In any case, it is quite clear that there are substantial impacts of both environmental and stochastic influences upon both life span and health span [99]. Discussion of environmental factors is beyond the scope of this minireview. Suffice it to say that there are likely to be a host of “gerontogens” [100] with the potential to modulate segmental and unimodal aspects of the pathobiology of aging. Cigarette smoking is a prime example [101]. With regard to stochastic factors, we must look to the work of colleagues who have demonstrated, in numerous publications over many decades, remarkable variations in life spans among highly inbred worms, flies, mice, hamsters, and rats, despite every effort to control the environments in which such organisms are aged. The most cogent example involved studies of C. elegans grown in liquid cultures with axenic medium [102]. One can imagine several distinct types of stochastic events to explain such observations. First, one can imagine stochastic variations in the epigenetic control of gene expression. Such a mechanism might explain, in part, recent experiments demonstrating correlations of the expression of a transgene for an inducible heat shock promoter/reporter with the longevity of cohorts of C. elegans [103]. There is also evidence of substantial “epigenetic drift” of gene expression within aging pairs of human identical twins [104,105]. The sirtuin family of histone deacetylases represents a potential causal link between epigenetic regulation, caloric restriction, and longevity in a number of organisms, including fruit flies [106]; moreover, inactivation of a member of the sirtuin family in mice causes phenotypes consistent with premature aging [107]. A second obvious candidate is somatic mutation, within both the nuclear [108] and mitochondrial [109] genomes. The latter is a particularly attractive idea, as the stochastic events could involve both the timing and specificity of the mutations and the events leading from the heteroplasmic to the homoplastic state. In that respect, certain classes of mitochondrial rearrangements leading to multiple replication origins might be more likely to evolve towards a homoplastic state [110]. In contrast to point mutations, which appear not to be major contributors to senescence [111], such rearranged mitochondrial DNA molecules might enjoy a selective replicative advantage over wild-type mitochondrial DNA. A third possibility could be related to what has been referred to in the microbial literature as “noise”—random fluctuations in gene expression; see, for example, [112]. Whatever the mechanism, these stochastic events, and the heritability studies mentioned above, diminish the power of genetic analysis to discover loci at which allelic variation modulates health span and life span. We already know a great deal about how the constitutional genome modulates the initiation and accumulation of somatic mutations, particularly nuclear mutations [34], but we know very little about how DNA sequences might set the stage for differing degrees of epigenetic variation and transcriptional or post-transcriptional noise.

## Opportunities for Discovering Genetic Contributions to Differential Rates of Physiological Declines in Middle-Aged Subjects

Human geneticists, most of whom are practicing medical geneticists, suffer from a biased ascertainment of their subjects. With the notable exception of studies of nonogenarians and centenarians discussed above, their subjects present themselves because of dysfunctions and never because of remarkably robust function. Centenarians, however, often have a variety of comorbidities. Moreover, given their extreme old age, it is not feasible to carry out longitudinal studies of the rates of change of specific physiological functions. An argument has been developed for a different approach to the discovery of allelic variants that are associated with unusual degrees of maintenance of structure and function during aging [113]. The suggested experimental design included a focus upon subjects in their early middle age, when early functional declines unfold, as predicted by the evolutionary biological theory of aging. Such subjects are typically free of comorbidities. In contrast to centenarians, there are vast numbers of such individuals and they are typically more compliant. They can be followed longitudinally for many years. Moreover, they are members of nuclear families, permitting sib-pair analysis and the use of multiple generations for the establishment of phase relationships of genetic markers. For many populations, there is a rich association of relevant clinical and pedigree information. Given sufficiently large cohorts of such individuals, one has the potential to detect individuals who are at the extreme ends of a statistical distribution of assays for a range of physiological functions. Those physiological assays must be highly sensitive, in order to identify individuals at the statistical extreme of exceptionally superior functioning. The assays should measure very specific physiological functions, functions that are not likely to be under highly polygenic controls. Ideally, they should also be relatively noninvasive, relatively inexpensive, relatively rapid, and not subject to major motivational influences that could impact peak performance. Multiple body systems should be interrogated, since an important null hypothesis to be tested, as noted in our Introduction, is the lack of tight coupling of rates of functional change among the various body systems. Given the availability of such assays, one would be in a position to carry out a genetic analysis on sib-pairs, starting with index cases from the extreme tails of the distributions. As argued by Risch and Zhang, the statistical power of such sib-pair studies would be enhanced by the selection of sibs showing extreme discordances [114]. The rapid technical advances that are being made in whole genome scans (see, e.g., http://www.perlegen.com/index.htm?whatweoffer/why_whole_genome.html) and statistical methodologies [115] should greatly facilitate the genetic analysis of exceptionally slow rates of human aging in various organ systems.

## Abbreviations

HGPS: Hutchinson-Gilford Progeria Syndrome
WS: Werner syndrome
